# A new fast filtering algorithm for a 3D point cloud based on RGB-D information

**DOI:** 10.1371/journal.pone.0220253

**Published:** 2019-08-16

**Authors:** Chaochuan Jia, Ting Yang, Chuanjiang Wang, Binghui Fan, Fugui He

**Affiliations:** 1 College of Mechanical & Electronic Engineering, Shandong University of Science and Technology, Qingdao, Shandong Province, China; 2 College of Electronics and Information Engineering, West Anhui University, Lu’an, Anhui Province, China; Nanjing University of Information Science and Technology, CHINA

## Abstract

A point cloud that is obtained by an RGB-D camera will inevitably be affected by outliers that do not belong to the surface of the object, which is due to the different viewing angles, light intensities, and reflective characteristics of the object surface and the limitations of the sensors. An effective and fast outlier removal method based on RGB-D information is proposed in this paper. This method aligns the color image to the depth image, and the color mapping image is converted to an HSV image. Then, the optimal segmentation threshold of the V image that is calculated by using the Otsu algorithm is applied to segment the color mapping image into a binary image, which is used to extract the valid point cloud from the original point cloud with outliers. The robustness of the proposed method to the noise types, light intensity and contrast is evaluated by using several experiments; additionally, the method is compared with other filtering methods and applied to independently developed foot scanning equipment. The experimental results show that the proposed method can remove all type of outliers quickly and effectively.

## Introduction

The 3D point cloud, due to its simplicity, flexibility and powerful representation capability, has become a new primitive representation for objects and has attracted extensive attention in many research fields, such as reverse engineering, 3D printing, archaeology, virtual reality, medicine and other fields [1–5]. Since a point cloud only needs to store the 3D coordinate values, it does not require the storage of the polygonal mesh connectivity [6] or topological consistency [7] such as triangle meshes. As a result, the manipulation of the point cloud can have better performance and lower overhead. These remarkable advantages make the research on manipulating point clouds become a hot topic.

**Fig 1:**
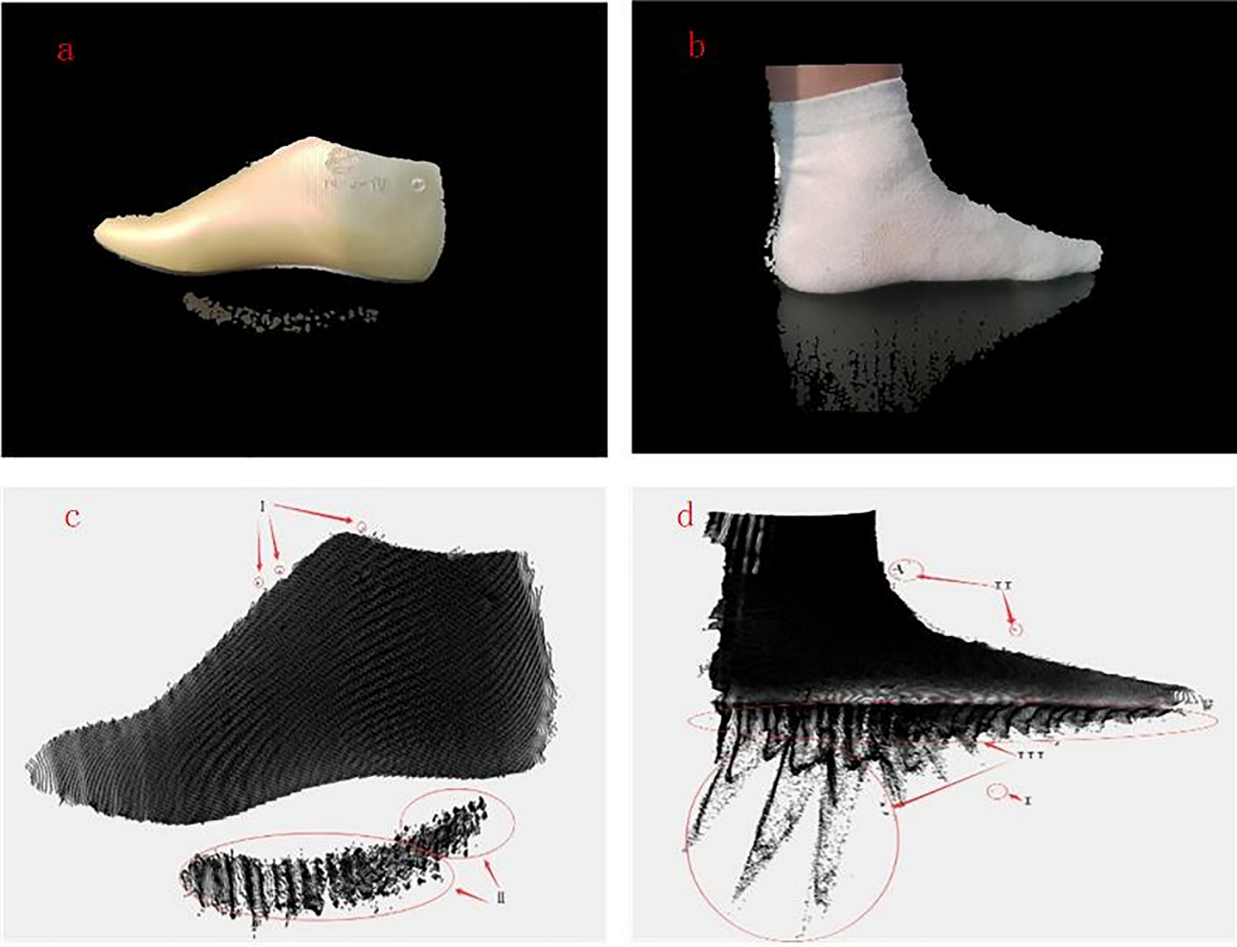
Original mapping image and point cloud. (a) and (b): Original mapping image;(c) and (d): Point cloud.

In recent years, with the development of optical components and computer vision technology and in addition to laser scanning sensors, low-cost RGB-D cameras have been rapidly developed, such as the Intel Realsense [8–10], Microsoft Kinect [11–13] and Astra; RGB-D cameras make it quite easy to obtain the point cloud of an object and have been widely used in many applications [14–17]. However, due to different view angles, light intensities, and reflective characteristics of object surfaces as well as the limitations of sensors [18], the point cloud data that are obtained by these RGB-D cameras will inevitably be affected by outliers that do not belong to the surface of the object. These outliers must be effectively removed in practical applications; otherwise, the subsequent processing of the point cloud, such as its measurement and surface reconstruction, will be seriously affected. These outliers can be divided into three types: I, sparse outliers; II, isolated clustered outliers; and III, non-isolated clustered outliers; which are shown in Fig 1. Therefore, performing the outlier removal operation on the original point cloud is the key step to obtaining accurate a point cloud for further processing. A new fast filtering algorithm for 3D point clouds is proposed in this paper. The main contributions of this paper are as follows: (i) The filtering problem of a 3D space is transformed into the filtering problem of a 2D plane. There is no need to calculate the geometric characteristics of the point cloud and design the judgment criterion in the 3D space. Therefore, the time consumption is greatly reduced. (ii) This filtering algorithm is a heuristic algorithm, and its implementation is simple. (iii) Compared with the existing filtering methods, it has a better filtering effect. (v) This method has good robustness to different types of noise.

The remainder of this paper is organized as follows. The related work is described in detail in section 2. Section 3 elaborates the depth image and RGB image alignment algorithm. In section 4, the proposed filtering methods are expanded, including point cloud data preprocessing, converting the RGB mapping image to an HSV image, image segmentation and extracting the point cloud. The experimental results are shown and discussed in section 5. Finally, conclusions are drawn in section 6.

## Related work

Outlier detection, which is an indispensable step in a 3D scanning system, is relatively complicated because outliers are disorganized and cluttered, have inconsistent densities, and the statistical distribution of these points is unpredictable. Thus, in recent years, many outlier detection methods for 3D point clouds have been proposed. The existing methods can be roughly summarized into four classifications as follows. First, there are neighborhood-based methods, which determine the new position of the sampling point using the similarity measurement between the sampling point and its neighborhood points [19]. As described in the literature, the similarity can be defined using the distance, angle, normal vectors, curvature and other feature information of points. The distance-base outlier detection method was designed by Kanishka et al. [20] and Gustavo et al. [21]. Kriegel et al. [22] proposed a novel method based on the angle between the difference vectors of a point to the other points in the neighborhood region. Bilateral filtering was originally proposed by Tomasi and Manduchi [23] and is a means of edge maintenance smoothing filtering; this approach has been extended to 3D point clouds based on the normal vectors and the intensity of points [24–26]. Wu et al. [27] designed a filtering algorithm based on the average curvature feature classification in which the traditional median and bilateral filtering algorithms are applied to different feature regions, respectively. Li et al. [28] put forward a denoising algorithm for point clouds based on the noise classification. The large-scale noise is removed by using statistical filtering and radius filtering, and then the small-scale noise is smoothed by using fast bilateral filtering. This algorithm can effectively maintain the geometric features of the scanned object; however, the correlation statistics parameters and radius parameters will have serious impacts on the filtering effect. Bradley Moorfield, et al. [29] first modified the normal vector by using bilateral filtering, and then the position of the samplingpoint was updated by using bilateral filtering with the modified normal vector. Zheng et al. [30] put forward a rolling normal filtering method. The weighted normal vector energy and weighted position energy function are applied to update the positions of points. This method can remove the different scales of geometric features well. An adaptive bilateral smoothing method is proposed by Li et al. [31]. The surface smoothing factor *δ*_*c*_ and the feature preserving factor *δ*_*s*_ are adaptively updated, and this method can effectively deal with the problems of feature shrinkage and over fairing. In conclusion, this kind of method has a good effect for the removal of isolated outliers and cannot obtain an ideal filtering effect on non-isolated outliers. The statistical-based methods are the second type of outlier detection methods, and they use the optimal standard probability distributions of data sets to identify outliers. Bayesian statistics were first employed to filter the point clouds by Jenke et al. [32]. They defined a measurement model that specified the probability distribution of the point cloud, and then three prior probabilities were defined to calculate the a posteriori probability, which is used for denoising while maintaining features. Patiño et al. [33] applied the Gaussian filter to reduce the directionality of high-density point clouds. A robust statistical framework is proposed for denoising point clouds by Kalogerakis et al. [34]. The normal vectors are corrected by using the statistical weights of the neighborhood points of the sampling point, and then the outliers are removed through the robust estimation of the curvature and normal vector. Lin et al. [35] proposed a feature preserving and noise filtering method based on the anisotropic Gaussian kernel. The adaptive anisotropic Gaussian kernel function combined with the bilateral filtering algorithm is constructed and applied to the denoising of scattered point clouds. The original sharp features of the point cloud model can be effectively maintained while removing the noise points using this method. However, a major limitation of statistical methods is the unpredictability of the probability distributions of data sets. Moreover, they do not work on non-isolated outliers such as type II and III outliers. Abdul et al. [36] proposed a statistical outlier detection method, in which the best-fit-plane is estimated based on the best possible and most consistent free distribution of outliers; then, outliers are detected and removed according to the normal vector and curvature of the best-fit-plane. This method has a good filtering effect on isolated outliers; however, it cannot achieve the ideal filtering effect on non-isolated outliers, and the computational complexity is also very high.

The density-based clustering methods that use unsupervised clustering technology to identify outliers are the third type of method. It is generally believed that small clusters with fewer data points will be recognized as outliers. Wang et al. [37] constructed statistical histograms according to the surface variation factor for each point, and the point cloud is divided into a normal cluster and an abnormal cluster by using the bi-means clustering method. Then, each point in the abnormal cluster is voted on by the normal points in its neighborhood; if majority of the vote consists of abnormal points, the point will be removed, and vice versa. This method has a good filtering effect on small scale isolated and non-isolated outliers; however, in the case of a large number of non-isolated outliers, it will not work well. Tao et al. [38] proposed an effective outlier detection and removal method that can preserve detailed features when removing the noise points. This method realizes noise data processing through two stages. In the first stage, the point cloud is classified into normal clusters, suspected clusters and outlier clusters by using density clustering, and in the second stage, the normal cluster points are determined to be suspected clusters through majority voting. This method can effectively remove the noise points and maintain the features of the model surface. However, this method needs to set the number of point clusters and density threshold, has high computational complexity and time consumption, and has little effect on dense non-isolated outliers. Yang et al. [39] proposed an outlier detection and removal method based on the dynamic standard deviation threshold of k-neighborhood density constraints. This method first extracts the target point cloud data using a pass-through filter and detects and removes invalid points. Then, it estimates the k-neighborhood density of the point cloud, dynamically adjusts the standard deviation threshold through the neighborhood density constraint, and sets different constraint methods for outlier detection for both the outer regions and inner regions. This method has a good filtering effect on point clouds with large differences in their density distributions; however, it has no effect on non-isolated outlier clusters such as type II and III outliers, and its computational complexity is relatively high. Model-based methods that learn a classifier from a known point cloud model are the last type of outlier detection method. Liu et al. [40] put forward the outlier detection method based on the support vector data description (SVDD) classification algorithm. This method first constructs a training data set and sets a confidence index for each point, and then a global SVDD classifier is built by using this training data set. Finally, the new sampling point is classified through the global classifier. Hido et al. [41] proposed a new statistical approach to detect outliers of high-dimensional data sets, which uses the ratio of training to test data as an outlier score. They trained a model based on the training data set without outliers, and then the outliers in the test data set are detected through this model. Model-based methods can achieve better filtering effects on the basis of knowing the training data set. However, the 3D point cloud models of objects are unpredictable in advance.

Although the above methods can remove the outlier noise points in 3D point clouds to a certain extent, all the algorithms mentioned above are directly applied to 3D point clouds, and their computational complexities are relatively high; therefore, it is difficult to apply them to actual scanning devices requiring real-time performance. Actually, the other auxiliary device and information except for the 3D position can be used to remove the outliers in the point cloud. Huynh et al. [42] proposed the outlier detection method based on the information of the object boundaries and shadows in a structured light 3D camera scanning system. This method can effectively remove all types of outliers. However, this method requires a projector to enhance the light. Thus, outlier noise point removal, which is the key step of a 3D scanning system, is still a hot topic with challenges. Therefore, a new fast filtering algorithm for 3D point clouds that are captured by RGB-D cameras is proposed in this paper.

## Depth image and color image alignment algorithm

**Fig 2:**
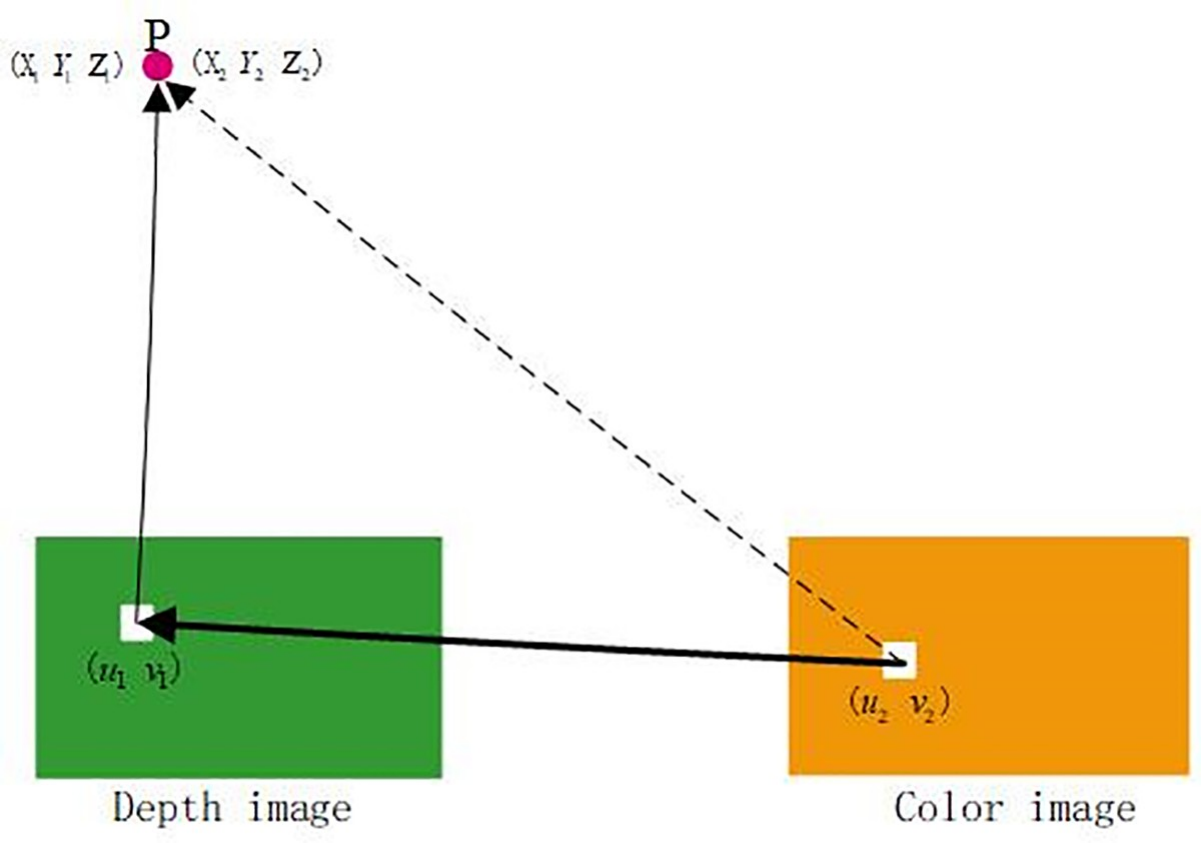
Alignment relationship diagram.

RGB-D cameras generally have two physical sensors: the infrared sensor that captures the depth image and the RGB sensor that captures the color image. Each sensor has its own two-dimensional pixel planar coordinate system and three-dimensional point cloud coordinate system. Assume that P is a point in the 3D space, and (*u*_1_,*v*_1_) and (*x*_1_,*y*_1_,*z*_1_), respectively, represent the 2D pixel coordinates and the 3D point cloud coordinates relative to the 2D pixel planar coordinate system and the 3D point cloud coordinate system on the depth sensor. Further, (*u*_2_,*v*_2_) and (*x*_2_,*y*_2_,*z*_2_) denote the 2D pixel coordinates and 3D point cloud coordinates for the RGB sensor, respectively. The relationship between (*u*_1_,*v*_1_) and (*x*_1_,*y*_1_,*z*_1_) can be formulated as Eq (1), and the relationship between (*u*_2_,*v*_2_) and (*x*_2_,*y*_2_,*z*_2_) can be formulated as Eq (2).

$$Z_{1}\left[\begin{array}{c} u_{1}\\ v_{1}\\ 1 \end{array}\right]=\left[\begin{array}{ccc} \frac{f_{1}}{dx_{1}} & 0 & u_{01}\\ 0 & \frac{f_{1}}{dy_{1}} & v_{01}\\ 0 & 0 & 1 \end{array}\right]\left[\begin{array}{c} X_{1}\\ Y_{1}\\ Z_{1} \end{array}\right]$$ (1)

$$Z_{2}\left[\begin{array}{c} u_{2}\\ v_{2}\\ 1 \end{array}\right]=\left[\begin{array}{ccc} \frac{f_{2}}{dx_{2}} & 0 & u_{02}\\ 0 & \frac{f_{2}}{dy_{2}} & v_{02}\\ 0 & 0 & 1 \end{array}\right]\left[\begin{array}{c} X_{2}\\ Y_{2}\\ Z_{2} \end{array}\right]$$ (2)

where *f*_1_,*dx*_1_,*dy*_1_,*u*_01_,*v*_01_,*f*_2_,*dx*_2_,*dy*_2_,*u*_02_,*v*_02_ are internal parameters of the depth sensor and RGB sensor. Suppose that the matrix *M*, which contains external parameters, represents the pose relationship between the depth sensor and RGB sensor, and the alignment relationship diagram is shown in Fig 2. The internal parameters and external parameters can be obtained by using the checkerboard calibration method [43]. Assume that these parameters are known. Then, Eq (3) can be derived from Fig 2.

$$\left[\begin{array}{l} X_{1}\\ Y_{1}\\ Z_{1}\\ 1 \end{array}\right]=M\left[\begin{array}{l} X_{2}\\ Y_{2}\\ Z_{2}\\ 1 \end{array}\right]=\left[\begin{array}{ll} R & t\\ 0 & 1 \end{array}\right]\left[\begin{array}{l} X_{2}\\ Y_{2}\\ Z_{2}\\ 1 \end{array}\right]$$ (3)

**Fig 3:**
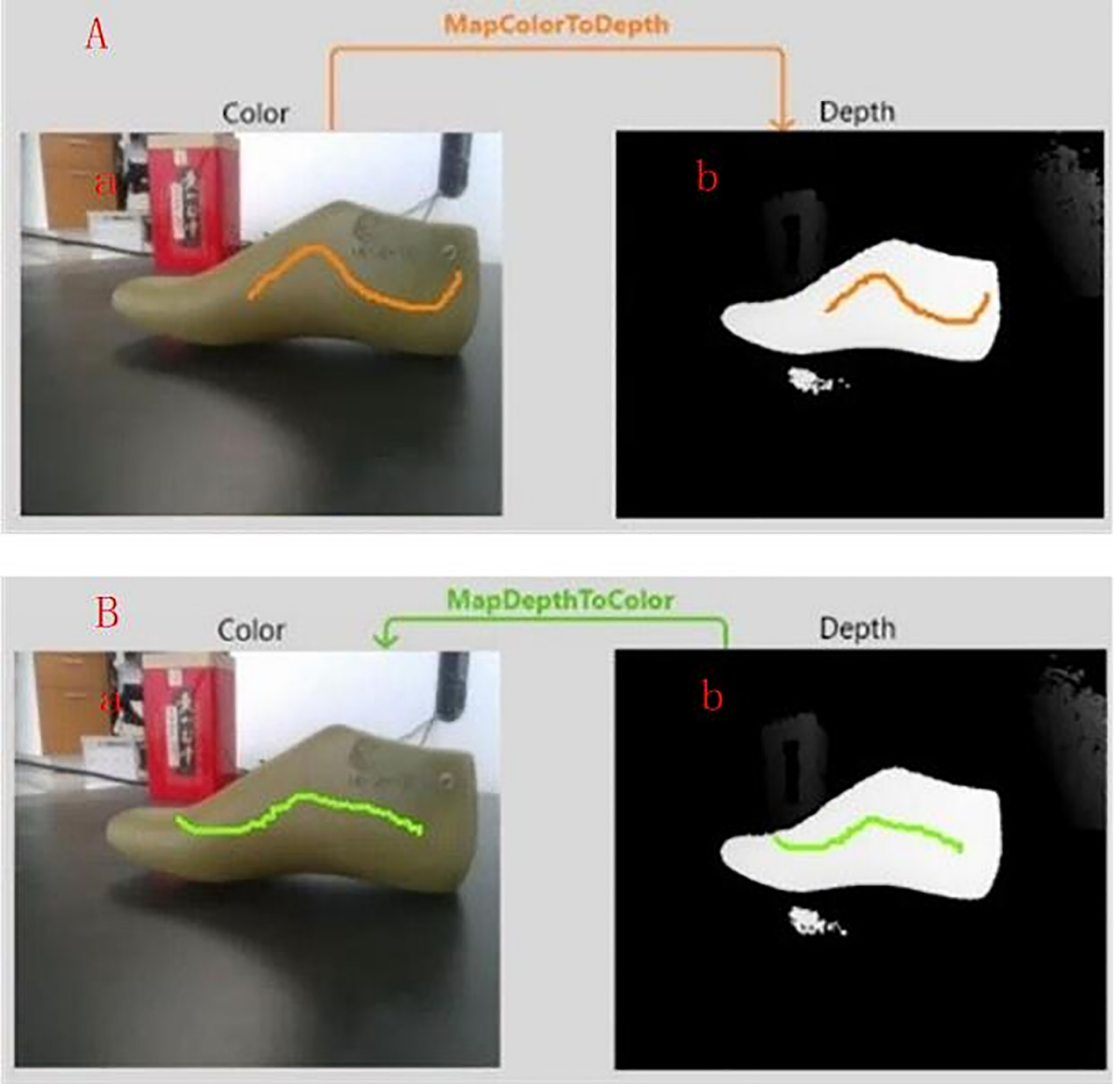
Alignment results. (A) color image alignment to depth image: (a) color image; (b) depth image; (B) depth image alignment to color image: (a) color image; (b) depth image.

Here, *R* denotes the rotation matrix, and *t* denotes the translation vector. Suppose that (*u*_1_,*v*_1_) denotes the arbitrary 2D sampling point on the depth image, and the corresponding spatial 3D point coordinate (*x*_1_,*y*_1_,*z*_1_) can be calculated by using formula (1). Then, (*x*_2_,*y*_2_,*z*_2_) can be obtained by using formula (3). Finally, (*u*_2_,*v*_2_) on the color image can be calculated by using formula (2). Therefore, the (*u*_1_,*v*_1_) and (*u*_2_,*v*_2_) that correspond to the same point in the 3D space are called a corresponding point pair. The alignment results are shown in Fig 3. In Fig 3(A), some consecutive points (orange points) on the color image are randomly selected, and then the corresponding points (orange points) are also selected on the depth image. In Fig 3(B), some consecutive points (green points) on the depth image are randomly selected, and then the corresponding points (green points) are also selected on the color image.

## Proposed method

**Fig 4:**
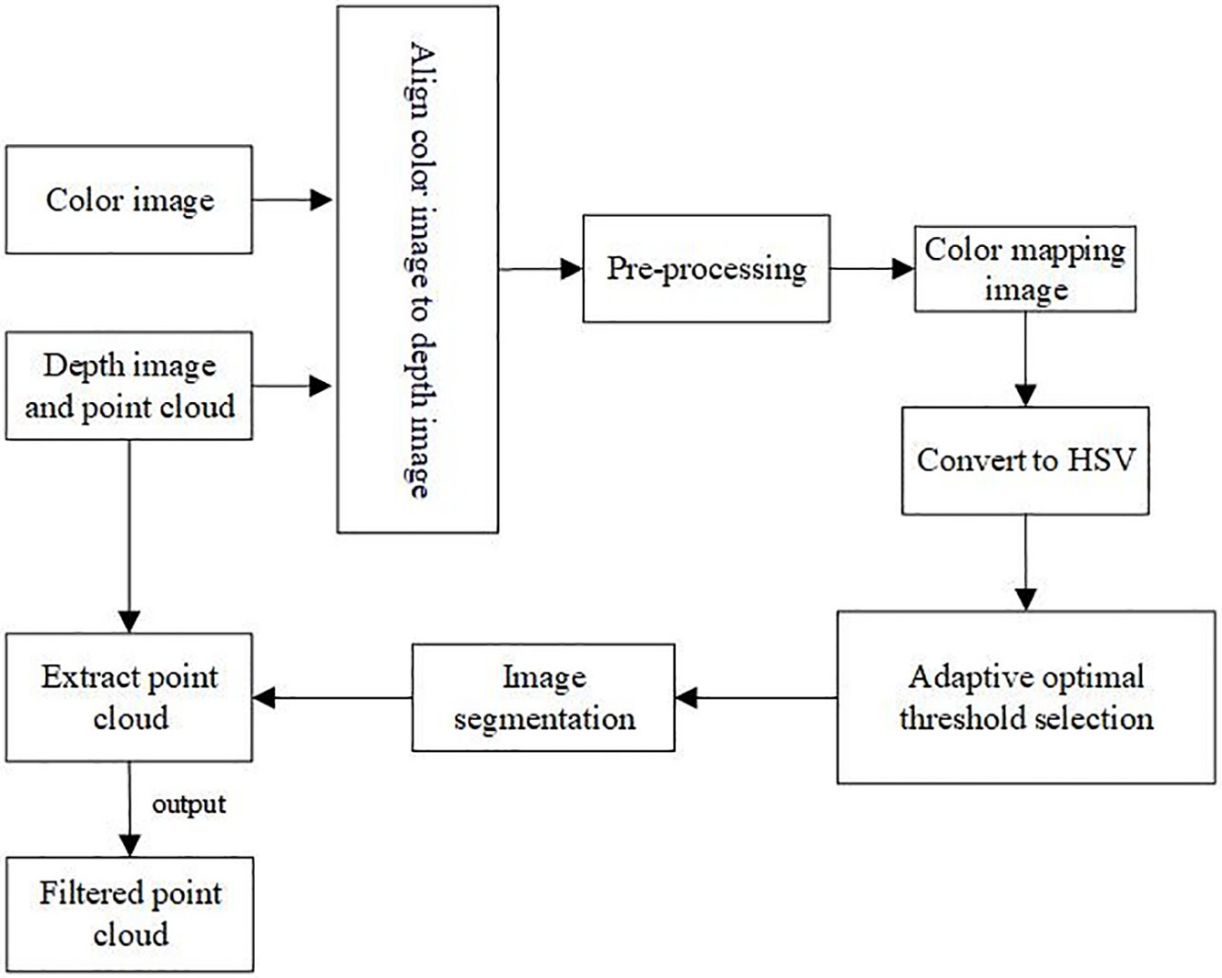
Overview of the proposed method.

The proposed 3D point cloud noise filtering method will be elaborated in detail in this section. First, the data that are captured by the cameras need to be preprocessed in order to facilitate the subsequent processing. Second, the color mapping image is converted to an HSV image. Then, the optimal threshold value is selected based on the V image for image segmentation. Finally, the target point cloud without noise points is extracted according to the segmentation results. The overview of the proposed method is shown in Fig 4.

### Preprocessing

The data acquisition device that is used in this paper is a Realsense SR300 camera that is produced by Intel, which can capture both color images, depth images and 3D point cloud data at the same time. Generally, the camera has a wide range of shooting angles and will synchronously acquire the data around the object that is scanned. To facilitate the subsequent processing, it is necessary to carry out coarse extraction of the target point cloud for the acquired point cloud.

The coarse extraction of the target point cloud roughly extracts the point cloud of the target from the data that contain a large number of noise points and background by using the 3D bounding box filtering method. First, the minimum (*x*_min_,*y*_min_,*z*_min_) and maximum (*x*_max_,*y*_max_,*z*_max_) values along the X, Y and Z directions are set, and then the RGB pixel and the 3D coordinate values of the points that are outside the range are set to zero. The 3D bounding box filtering method is formulated as follows.

$$\begin{array}{l} P=\left\{p_{i}|Min\leq p_{i}\leq Max\right\}=\\ \;\left\{ \begin{array}{l} x_{\min}\leq x_{i}\leq x_{\max}\\ y_{\min}\leq y_{i}\leq y_{\max}\\ z_{\min}\leq z_{i}\leq z_{\max} \end{array}\right. \end{array}$$ (4)

### Color mapping image converted to an HSV image

The color mapping image is still an RGB image, which is sensitive to the light intensity. Therefore, it needs to be converted to an HSV image, which is robust to the light intensity. However, the RGB image should be normalized to the range of [0,1] before the conversion. The conversion formulas are shown as follows.

V=max(R,G,B) (5)

$$S=\left\{ \begin{array}{l} \frac{V-\min(R,G,B)}{V}if\;V\neq 0\\ \;0\;\mathrm{otherwise} \end{array}\right.$$ (6)

$$H=\left\{ \begin{array}{l} \frac{60\times (G-B)}{V-\min(R,G,B)}\;if\;V=R\\ 60\times (2+\frac{\left(B-R\right)}{V-\min(R,G,B)})\;if\;V=G\\ 60\times (4+\frac{\left(R-G\right)}{V-\min(R,G,B)})\;if\;V=B \end{array}\right.$$ (7)

H=H+360,ifH<0 (8)

According to Eqs (5)–(8), the RGB image can be divided into three images, which are the V image, the S image and the H image.

### Optimal threshold selection algorithm

Image segmentation is a routine process in which the image is divided into several disjointed or non-overlapping regions, and then the target is detected and separated from the background [44–46]. After the image is segmented, the segmented objects can be identified and classified. The image segmentation in this paper seeks to separate the target from the noises and then to extract the point cloud of the target. The optimal threshold method is used to segment the target from the background. There are many methods to select the optimal threshold, but according to the different image types, the adaptive ability of each algorithm is also different. Here, the threshold of V is adaptively determined by adopting the Otsu algorithm [47–48], which is based on the principle of the maximum variance.

Assume that the grayscale of the V image is divided into L grades for a given image. The pixel number is *n*_*i*_ when the gray value is *i*. Therefore, the total pixel numbers and the probability for the grayscale image are shown as follows:

$$N=\sum_{i=1}^{L}n_{i}$$ (9)

$$p_{i}=n_{i}/N,\;p_{i}\geq 0,\;\sum_{i=1}^{L}p_{i}=1$$ (10)

The initial K is chosen to divide all pixels of this image into two groups, *C*_0_ = {1~*K*} and *C*_1_ = {*K*+1~*L*}. Then, their probabilities and mean values are shown as follows:

$$\omega_{0}=\mathrm{Pr}(C_{0})=\sum_{i=1}^{k}p_{i}=\omega (k)$$ (11)

$$\omega_{1}=\mathrm{Pr}(C_{1})=\sum_{i=k+1}^{L}p_{i}=1-\omega (k)$$ (12)

$$\mu_{0}=\sum_{i=1}^{k}i\mathrm{Pr}(i|C_{0})=\sum_{i=1}^{k}ip_{i}/\omega_{0}=\mu (k)/\omega (k)$$ (13)

$$\mu_{1}=\sum_{i=k+1}^{L}i\mathrm{Pr}(i|C_{1})=\sum_{i=k+1}^{L}ip_{i}/\omega_{1}=\frac{u_{T}-\mu (k)}{1-\omega (k)}$$ (14)

Here, $\mu_{T}=\mu (L)=\sum_{i=1}^{L}ip_{i}$, which is the average of the gray values of the image; $\mu (k)=\sum_{i=1}^{k}ip_{i}$, which is the average of the gray values with threshold K; and *ω*_0_*μ*_0_+*ω*_1_*μ*_1_ = *μ*_*T*_, *ω*_0_+*ω*_1_ = 1.

The variance between the two groups is

$$\begin{array}{l} \sigma_{}^{2}(k)=\omega_{0}(\mu_{0}-\mu_{T}{)}^{2}+\omega_{1}(\mu_{1}-\mu_{T}{)}^{2}\\ \;=\frac{{\left[\mu_{T}\omega (k)-\mu (k)\right]}^{2}}{\omega (k)[1-\omega (k)]} \end{array}$$ (15)

Obviously, the grayscale histogram will be divided into two groups using the optimal threshold, which is calculated by maximizing the variances of the two groups. When K changes from 1 to L, the K that maximizes Eq (15) is the optimal segmentation threshold *k*_*opt*_. The V image is regarded as the image to be segmented in this paper, and then the optimal segmentation threshold of the V image can be obtained.

### Image segmentation

Based on the V image from the Otsu algorithm, *k*_*opt*_, the best threshold of V is obtained. Then, the projection image is converted into a binary image using the optimal threshold. The image segmentation can be formulated as follows.

$$V_{binary}(x,y)=\left\{ \begin{array}{ll} 0 & if\;V(x,y)<k_{opt}\\ 1 & others \end{array}\right.$$ (16)

Here, *V*_*binary*_ represents the segmented binary image, and (*x*,*y*) denotes the location of pixels. However, some holes may appear in the binary image; therefore, hole filling should be conducted by applying morphological dilation and erosion on the *V*_*binary*_ image.

### Extracting target point cloud

Since 0 represents a noise point or background point, 1 represents the target point in the binary image that is obtained by image segmentation. Therefore, the target point cloud without noise points can be obtained by using the *V*_*binary*_ image.

## Experimental results and analysis

### Different perspective

**Fig 5:**
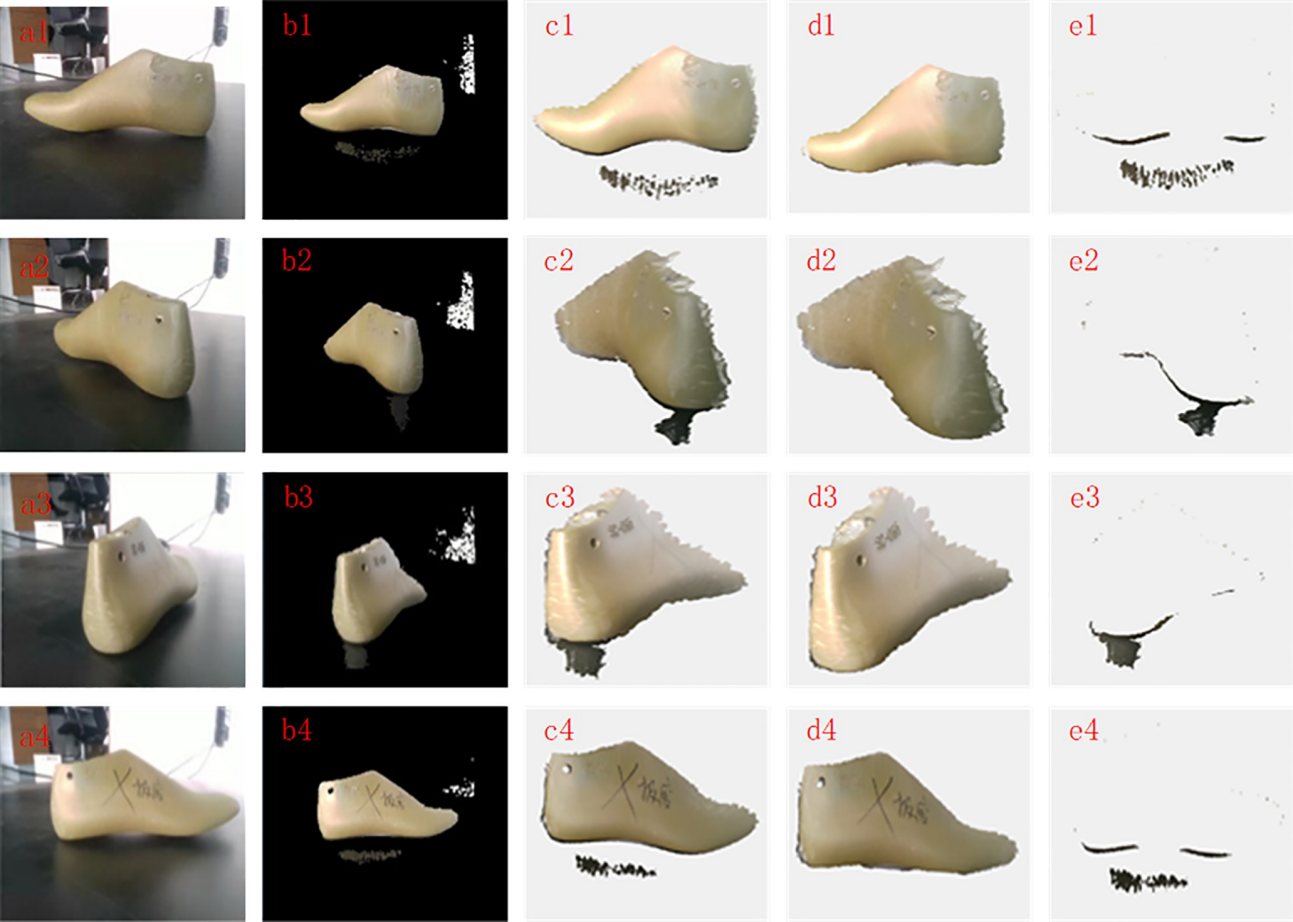
**Point cloud filtering results with different types of noise:** (a1~a4): RGB images of View 1, View 2, View 3 and View 4; (b1~b4): RGB mapping images of View 1, View 2, View 3 and View 4; (c1~c4): Original point cloud with color of View 1, View 2, View 3 and View 4; (d1~d4): Point cloud with color after filtering of View 1, View 2, View 3 and View 4; (e1~e4): Removed point cloud with color of View 1, View 2, View 3 and View 4.

In this experiment, different perspectives will capture different surface point clouds that contain different types of noise due to the different incident and reflection angles of light. Therefore, in order to verify the robustness of the proposed method to different types of noise, this method is applied to point clouds with different types of noise. The experimental results are shown in Fig 5. It can be clearly seen from the original point cloud with color that the isolated outliers are mainly included in View 1 and View 3, while the non-isolated outliers are mainly included in View 2 and View 4. From the point cloud with color after filtering, it can be found that the proposed filtering method can not only remove the isolated outliers but also eliminate the non-isolated outliers. Meanwhile, it was found from the removed point cloud with color that some valid points were removed by mistake, which are mainly concentrated near the contact surface of the object and the platform because of the small contrast on the contact surface. Since the number of these valid points that are removed is small and they cannot change the contour of the scanned object, this approach is acceptable in engineering. Therefore, the proposed filtering method has good robustness to different types of noise.

### Different light intensity

**Fig 6:**
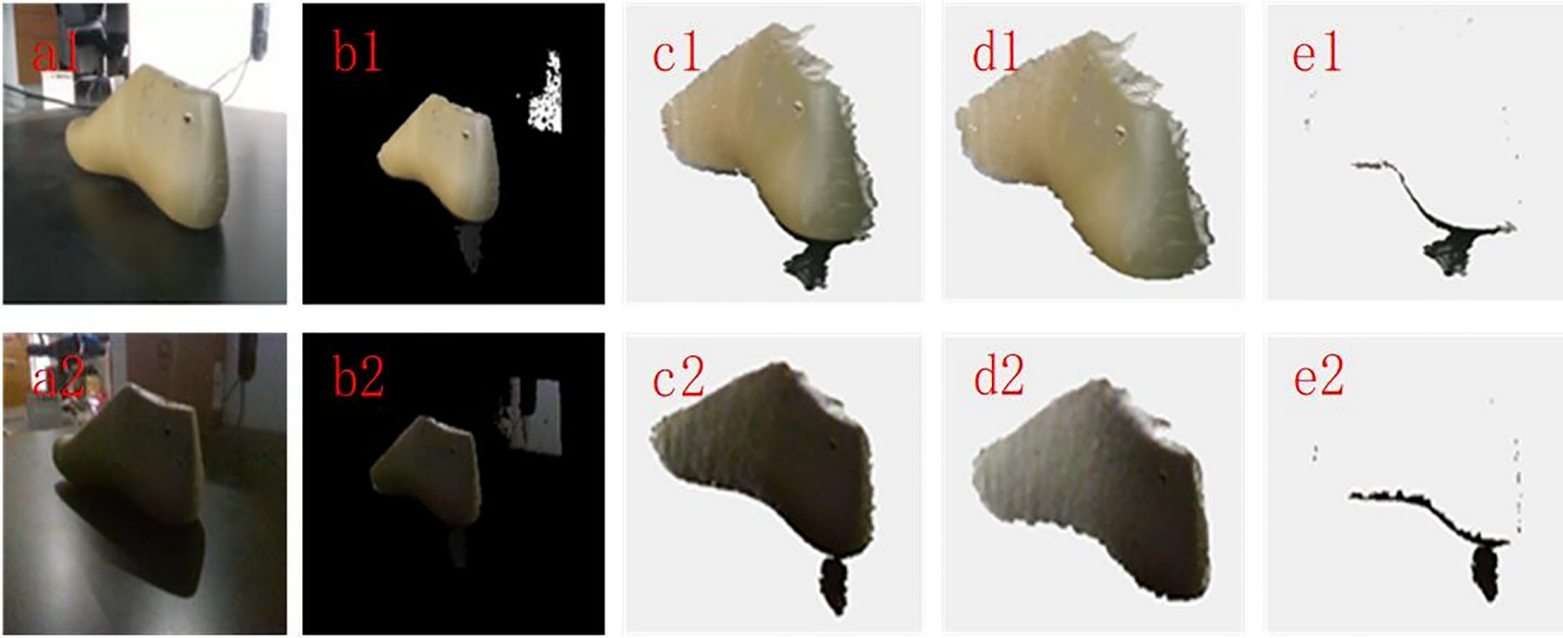
**Point cloud filtering results with different different light intensities:** (a1,a2): RGB images of Strong light and Weak light; (b1,b2): RGB mapping images of Strong light and Weak light; (c1,c2): Original point cloud with color of Strong light and Weak light; (d1,d2): Point cloud with color after filtering of Strong light and Weak light; (e1,e2): Removed point cloud with color of Strong light and Weak light.

Different light intensities will cause the RGB pixel information of the color image to dramatically change, which will affect the effective segmentation of an image. Therefore, in order to verify the robustness of the proposed filtering method to different light intensities, the method is applied to point cloud filtering under two different lighting conditions, which are strong light and weak light. The experimental results are shown in Fig 6. The two RGB images in the table were captured under strong and weak light conditions. From these RGB images, it can be clearly found that the RGB pixel values of the two images have dramatically changed. However, from the point cloud with color after filtering, it can be found that the proposed filtering method can not only remove the noises points under the strong light condition but also remove the noise points under the weak light condition. It was also found from the removed point cloud with color that some valid points were removed by mistake, and the reason is the same as above. Since the number of these valid points that are removed is small and they cannot change the contour of the scanned object, this removal is acceptable in engineering. Therefore, the proposed filtering method has good robustness to different light intensities.

### Different reflective surfaces

**Fig 7:**
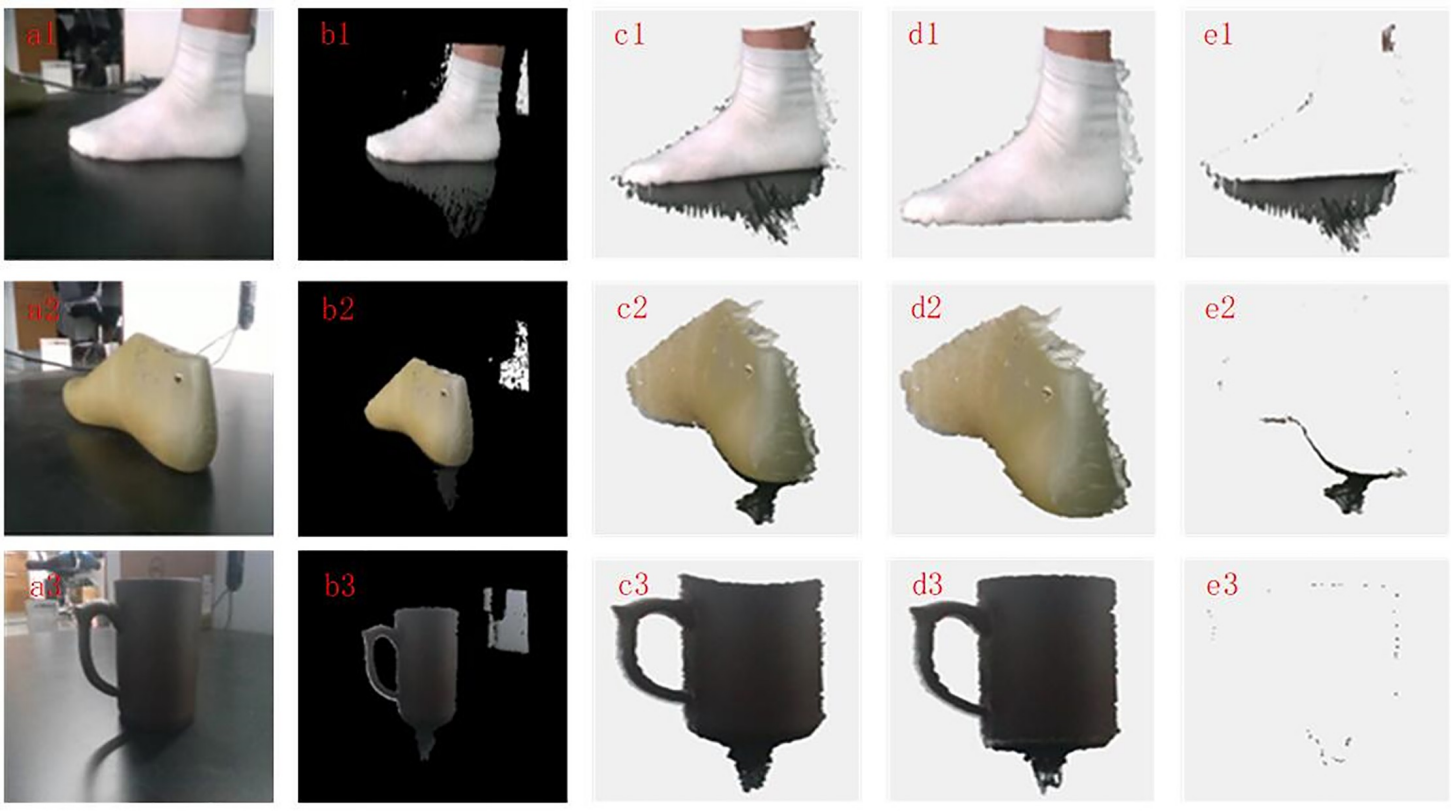
**Point cloud filtering results with different reflective surfaces:** (a1~a3): RGB images of Reflective surface 1, Reflective surface 2 and Reflective surface 3; (b1~b3): RGB mapping images of Reflective surface 1, Reflective surface 2 and Reflective surface 3; (c1~c3): Original point cloud with color of Reflective surface 1, Reflective surface 2 and Reflective surface 3; (d1~d3): Point cloud with color after filtering of Reflective surface 1, Reflective surface 2 and Reflective surface 3; (e1~e3): Removed point cloud with color of Reflective surface 1, Reflective surface 2 and Reflective surface 3.

The proposed filtering method is mainly based on image contrast segmentation. Therefore, the method is applied to different objects with different reflective surfaces. The experimental results are shown in Fig 7. Three objects with different reflective surfaces are included in the table. It can be seen that the contrast of Reflective surface 1 is the highest, that of Reflective surface 2 is in the middle, and that of Reflective surface 3 is the smallest. From the point cloud with color after filtering, the proposed filtering method can remove the noise points in Reflective surface 1 and Reflective surface 2. However, the proposed method will not work properly for Reflective surface 3 because the contrast of Reflective surface 3 is too small to complete the correct segmentation between the object and the platform. Therefore, the proposed method can obtain a good effect when the reflective surface of the object is bright, but it will not work properly when the reflective surface of the object is dark.

### Comparing different filtering algorithms

**Fig 8:**
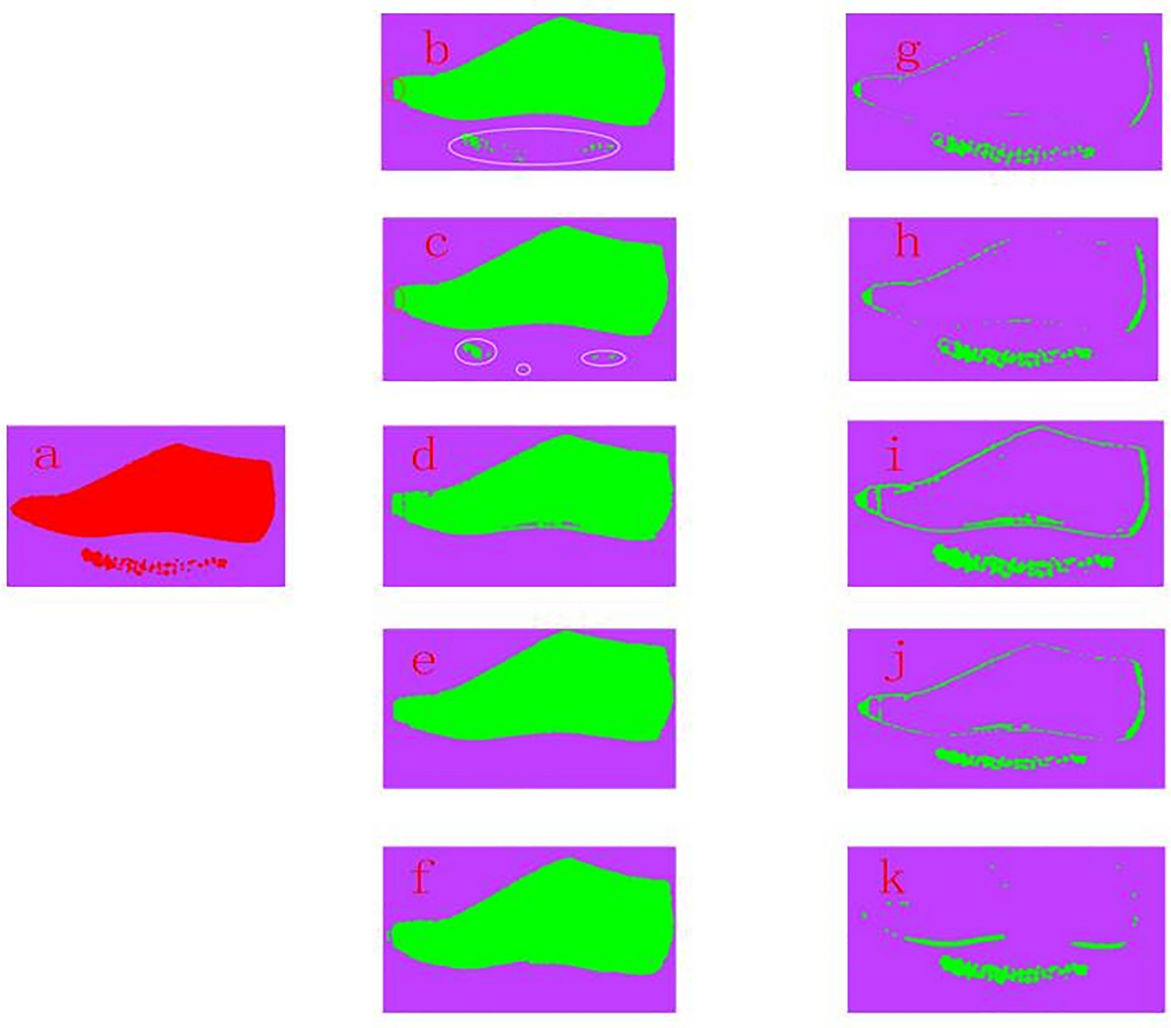
Comparison results of different filtering methods for view 1. (a) Original point cloud. (b)~(f) Point cloud after filtering: (b) SOR, (c) ROR, (d) Wang [37], (e) Tao [38], and (f) Proposed. (g)~(k) Removed point cloud: (g) SOR, (h) ROR, (i) Wang[37], (j) Tao[38], and (k) Proposed.

**Fig 9:**
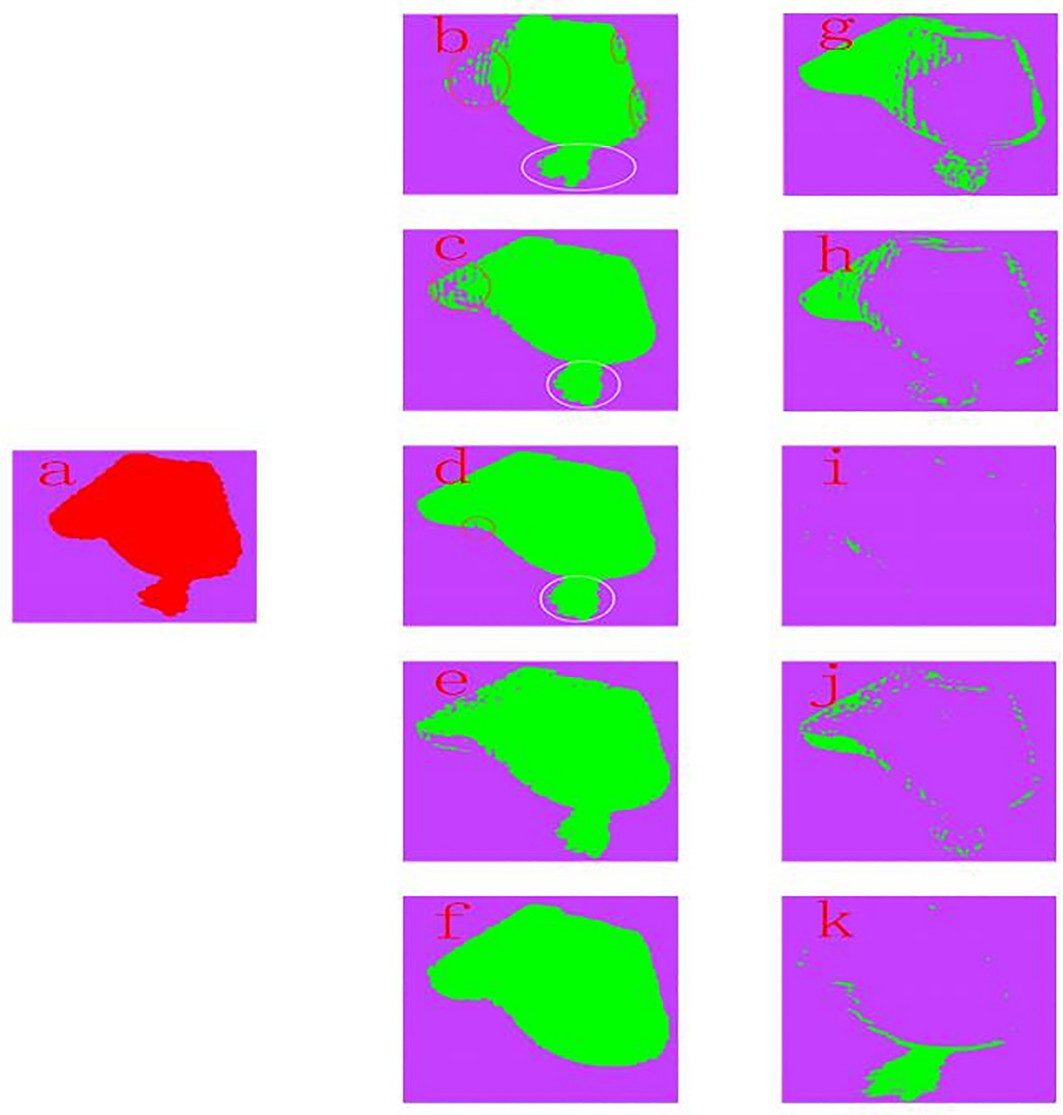
Comparison results of different filtering algorithms for view 2. (a) Original point cloud. (b)~(f) After Filtered point cloud: (b) SOR, (c) ROR, (d) Wang [37], (e) Tao [38], and (f) Proposed. (g)~(k)Removed point cloud: (g) SOR, (h) ROR, (i) Wang[37], (j) Tao[38], and (k) Proposed.

To further verify the effectiveness and real-time performance of the proposed method, this method is compared with statistical outlier removal (SOR) and radius outlier removal (ROR), which are in the point cloud library (PCL), and the methods in [37] and [38]. In the experiments, there are some parameters that need to be predefined in the SOR method, which are the size *k* of the k-nearest neighbor and the distance standard deviation *σ*, and these two parameters need to be determined through multiple tests. The filtering effect is good when *σ* = 0.5 and *k* = 15. In the ROR method, the search radius *r* and the number of interior points *num* need to be set. After much experimenting, the filtering effect is good when *r* = 0.002*m* and *num* = 12. The parameters of the filtering method in the literature [37] and [38] are set with respect to the corresponding literatures. There is only one parameter that needs to be set in the proposed method, which is the area threshold *s*_*th*_,and it is easy to set according to the total number of pixels of the scanned object in an image. When the scanned object is the last shoe, *s*_*th*_ is set 5000. The abovementioned five filtering methods are applied to the point cloud of the last shoe, which is captured from two perspectives: view 1 and view 2. The experimental results are shown in Fig 8 and Fig 9, and the time consumptions of the different methods are recorded in Table 1 and Table 2. Fig 8 shows the comparison results of the different filtering methods for view 1, and Fig 8(A) is the original point cloud that contains the isolated outliers. From Fig 8(B) and 8(C), which are the SOR and ROR results, respectively, the points in the white circle are noise points that are not successfully removed. Meanwhile, some valid points in the red circle have been removed by mistake. It can be seen that no matter how the relevant parameters are adjusted, these two methods cannot completely remove the isolated outlier clusters. From Fig 8(D) and 8(E), which are the results of Wang [37] and Tao [38], respectively, although these two methods can completely remove isolated outlier clusters, they have removed many valid points by mistake (Fig 8(G) and Fig 8(H)), which affects the surface of the object. Fig 8(F) is the result of the proposed method, which can remove all isolated outlier clusters, but some valid points will also be removed by mistake. The size of the noise points, the size of points removed, the size of valid noise points removed, the size of points mistakenly removed and the run times are recorded in Table 1. From Fig 8(I) to 8(K) and Table 1, it can be seen that in the case of completely removing the isolated outlier clusters, the number of valid points that are removed by the proposed method that takes the shortest time is minimal and the size of points mistakenly removed is smallest. Fig 9 shows the comparison results of different filtering methods for view 2, and the size of the noise points, the size of points removed, the size of valid noise points removed, the size of points mistakenly removed and the run times are recorded in Table 2. Fig 9(A) is the original point cloud that contains the non-isolated outliers. From Fig 9(B) and 9(C), which are the SOR and ROR results, respectively, these two methods have been shown to not work properly for non-isolated outlier clusters. From Fig 9(D) and 9(E), which are the results of Wang [37] and Tao [38], respectively, these two methods have also been shown to not work properly for non-isolated outlier clusters. However, the proposed method can completely remove the non-isolated outlier clusters from Fig 9(F). From Fig 9(I) to 9(K) and Table 2, the same conclusion as mentioned above can be obtained. In summary, the proposed method has good robustness to different types of noise, and it can be applied to projects that require high real-time performance since it has extremely short time consumption.

**Table 1:** View 1 point cloud filtering comparison results for different filtering methods.

Point cloud	Method	Size of noise points	Size of points removed	Size of valid noise points removed	Size of points mistakenly removed	Time(s)
View 1	SOR	2553	2317	1758	559	5.2765
	ROR		2270	1684	586	2.7582
	Wang [37]		3663	2553	1110	13.0025
	Tao [38]		3232	2553	679	12.3462
	Proposed		2836	2553	283	**0.6454**

**Table 2:** View #2 point cloud filtering comparison result between different filtering algorithms.

Point cloud	Method	Size of noise points	Size of points removed	Size of valid noise points removed	Size of points mistakenly removed	Time(s)
View 1	SOR	2887	4976	484	4492	4.2765
	ROR		1962	185	1777	1.8237
	Wang [37]		76	5	71	5.2453
	Tao [38]		2235	116	2119	9.3462
	Proposed		3047	2887	160	**0.6106**

### Supplementary experiment

**Fig 10:**
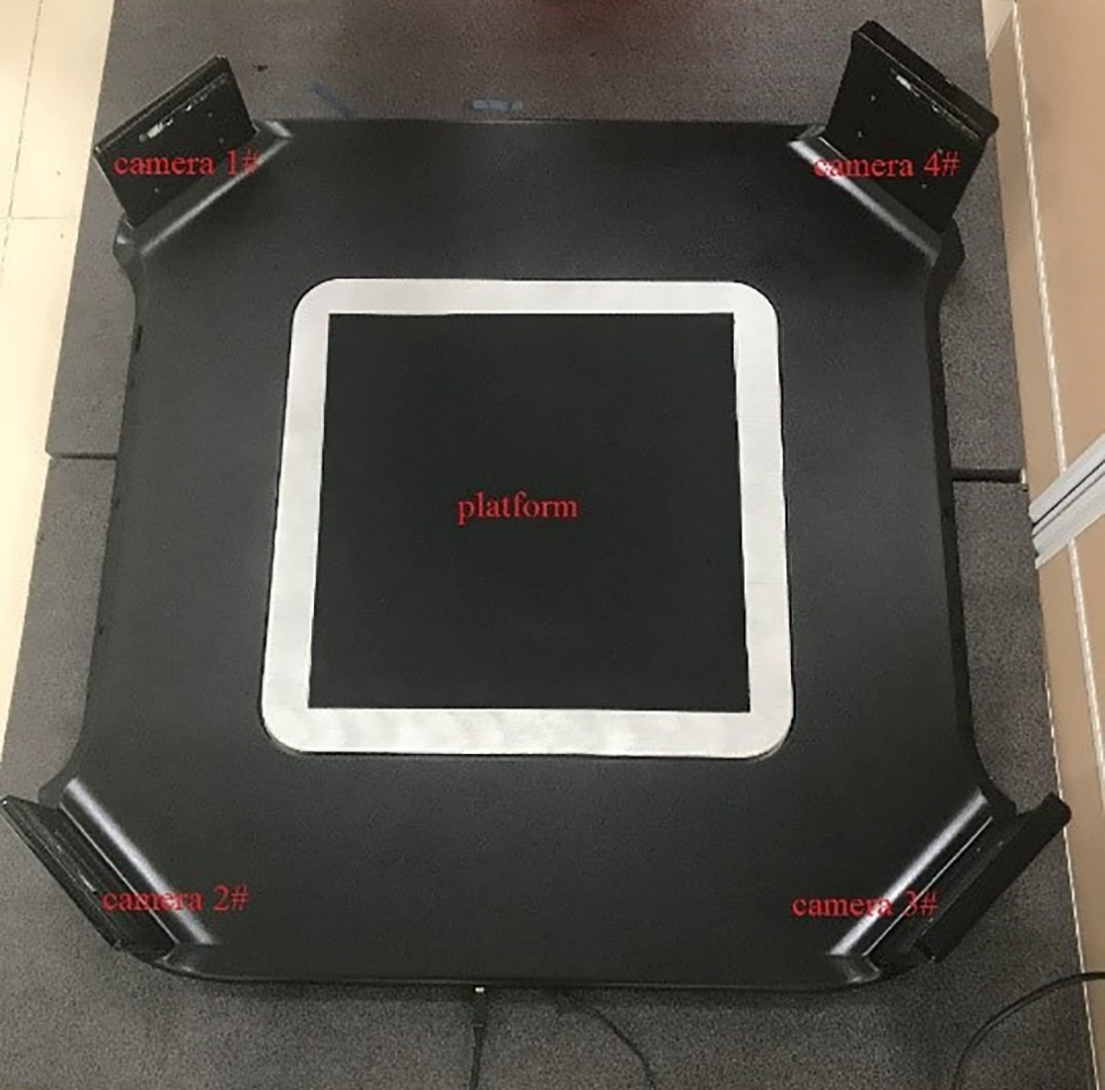
Overview of foot scanning equipment.

**Fig 11:**
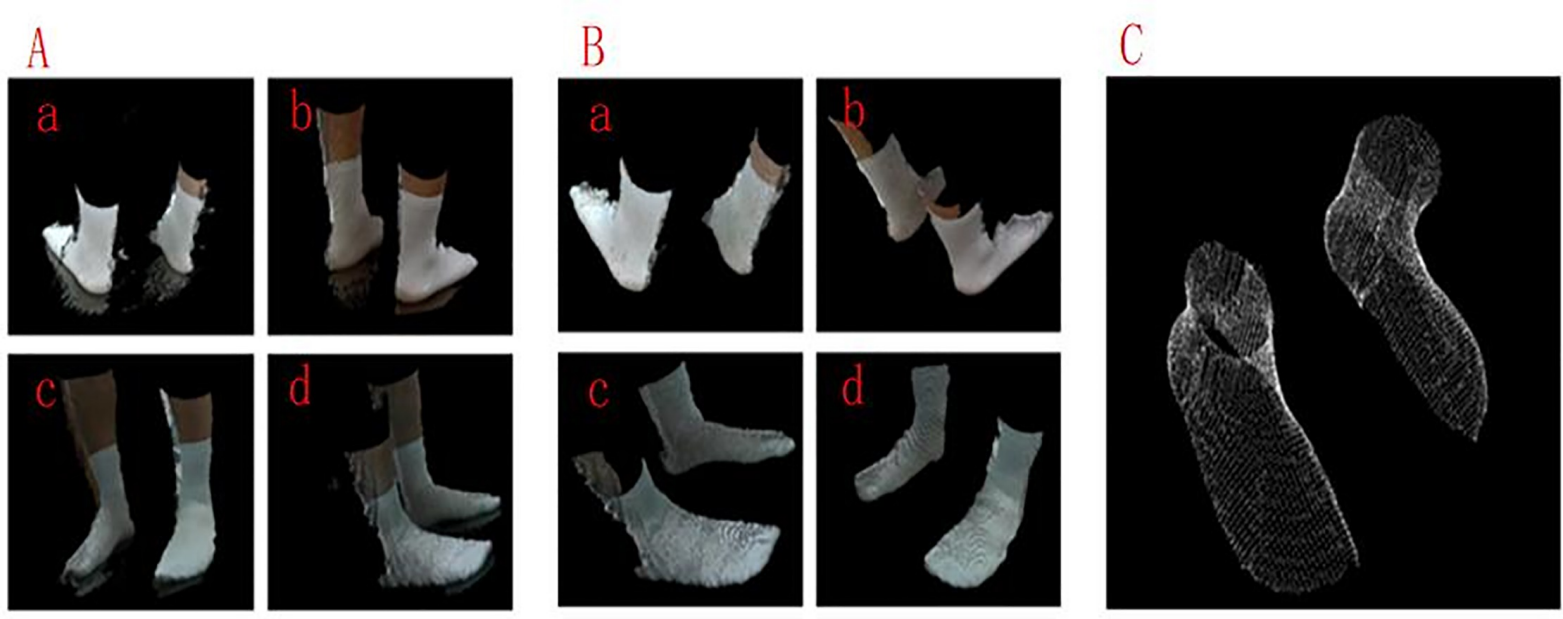
**Scanning result:** (A) Original point clouds: (a) Original point cloud of camera 1#, (b) Original point cloud of camera 2#,(c) Original point cloud of camera 3#, (d) Original point cloud of camera 4#, (B) Filtered point clouds: (a) Filtered point cloud of camera 1#, (b) Filtered point cloud of camera 2#,(c) Filtered point cloud of camera 3#, (d) Filtered point cloud of camera 4#, and (C) Complete point cloud model.

To verify the validity and practicability of the proposed method, the proposed filtering method is applied to the independently developed foot scanning equipment. Four SR300 cameras, which are labeled as camera #1, camera #2, camera #3 and camera #4, are located vertically at the four corners and point to the center of the platform. The overview of the equipment is shown in Fig 10. When the object is placed on the platform, the system can capture the object from four different perspectives. The proposed method was applied to each camera to filter the noise points and remove the background, and the four filtered point clouds are transformed into a unified coordinate system to achieve rough matching. Then, the iterative closest point (ICP) algorithm is used to achieve the fine matching of two adjacent point clouds. Finally, a complete 3D point cloud model that provides accurate data support for subsequent processing, such as the reconstruction and feature parameter computations, is obtained. The scanning result is shown in Fig 11. From Fig 11(A) and the original point cloud that is captured by each camera contains many different types of noise points. As seen from Fig 11(B), all noise points have been successfully removed from the original point cloud by using the proposed filtering method. The complete point cloud model that is the closest to the real shape of the object is shown in Fig 11(C).

## Conclusions

A fast and robust 3D point cloud filtering method has been proposed in this paper to effectively remove all types of outliers from a scanned point cloud, which is captured by a scanning system consisting of an RGB camera and a depth camera. This method segmented the mapping image, modifying from an RGB image to a depth image, and extracted the point cloud of a target object according to the segmentation result, which removes all outlier noise. As various experimental studies have proven, the proposed method has several advantages, as follows: (i) The 3D point cloud filtering problem is transformed into a 2D image segmentation problem, which contributes the dimensionality reduction. (ii) The time consumption of the proposed method is short enough for real-time point cloud filtering, which provides the possibility for 3D scanning to realize real-time processing, such as the foot scanning system mentioned above. (iii) The number of valid points that are removed from the surface of the scanned object is minimal, while the outlier noises are completely removed. (iv) This method is very robust to the light intensity and viewing angle. (v) This method has good robustness to different types of noise. However, this method also has some limitations, as follows: (i) this method is only applicable to scanning systems that contain both an RGB camera and a depth camera and (ii) this method is only applicable to the application scenarios where the scanned object is in stark contrast to the background platform. To improve the filtering performance of this method, how to identify the mistakenly removed point clouds will be studied in the future.

## Supporting information

S1 Fig Original mapping image and point cloud. (a) original mapping image of shoe last.
(TIF)

S2 Fig Original mapping image and point cloud. (b) original mapping image of foot.
(TIF)

S3 Fig Original mapping image and point cloud. (c) Point cloud of shoe last.
(TIF)

S4 Fig Original mapping image and point cloud. (d) Point cloud of foot.
(TIF)

S5 Fig Alignment relationship diagram. (TIF)

S6 Fig Alignment results. (a) color image alignment with respect to the depth image.
(TIF)

S7 Fig Alignment results. (b) depth image alignment with respect to the color image.
(TIF)

S8 Fig Overview of the proposed method. (TIF)

S9 Fig Comparison results of different filtering methods for view 1. (TIF)

S10 Fig Comparison results of different filtering algorithms for view 2. (TIF)

S11 Fig Overview of foot scanning equipment. (TIF)

S12 Fig **Scanning result:** (a) Original point clouds.
(TIF)

S13 Fig **Scanning result:** (b) Filtered point clouds.
(TIF)

S14 Fig **Scanning result:** (c) Complete point cloud model.
(TIF)

S1 Appendix All raw point cloud datasets. (RAR)
