# Supplementary figures and images for: A new fast filtering algorithm for a 3D point cloud based on RGB-D information
Source: PLoS One. 2019 Aug 16;14(8):e0220253. doi: 10.1371/journal.pone.0220253 (PMC6697356; doi:10.1371/journal.pone.0220253)

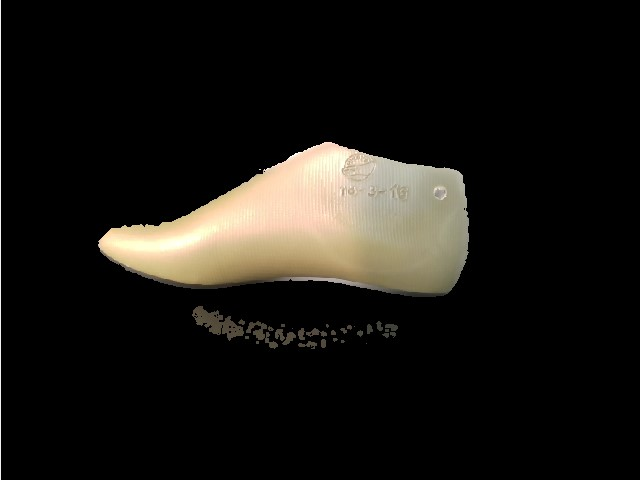

Supplement: S1 Fig — (a) original mapping image of shoe last. (TIF) [file pone.0220253.s001.tif]

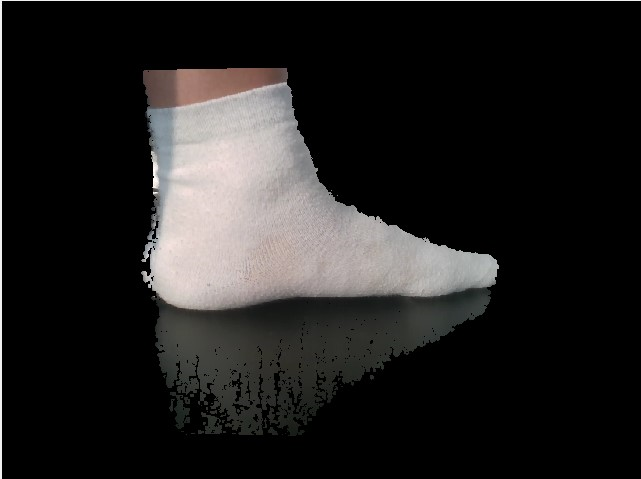

Supplement: S2 Fig — (b) original mapping image of foot. (TIF) [file pone.0220253.s002.tif]

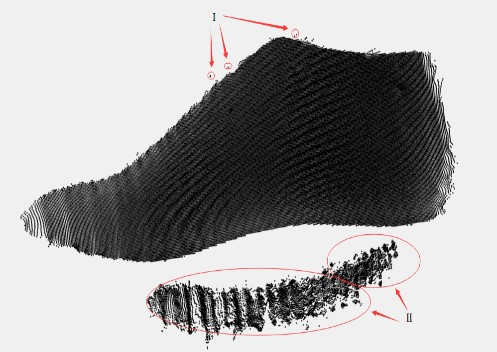

Supplement: S3 Fig — (c) Point cloud of shoe last. (TIF) [file pone.0220253.s003.tif]

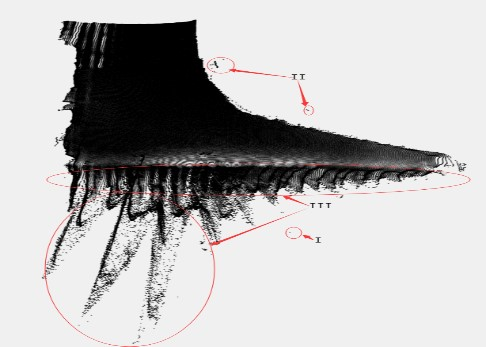

Supplement: S4 Fig — (d) Point cloud of foot. (TIF) [file pone.0220253.s004.tif]

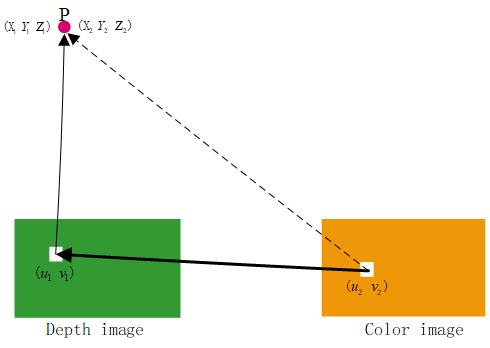

Supplement: S5 Fig — (TIF) [file pone.0220253.s005.tif]

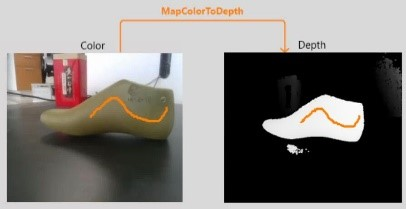

Supplement: S6 Fig — (a) color image alignment with respect to the depth image. (TIF) [file pone.0220253.s006.tif]

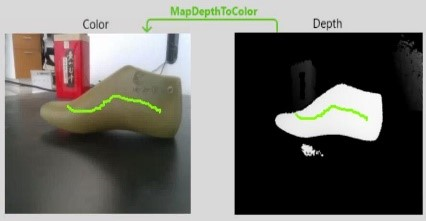

Supplement: S7 Fig — (b) depth image alignment with respect to the color image. (TIF) [file pone.0220253.s007.tif]

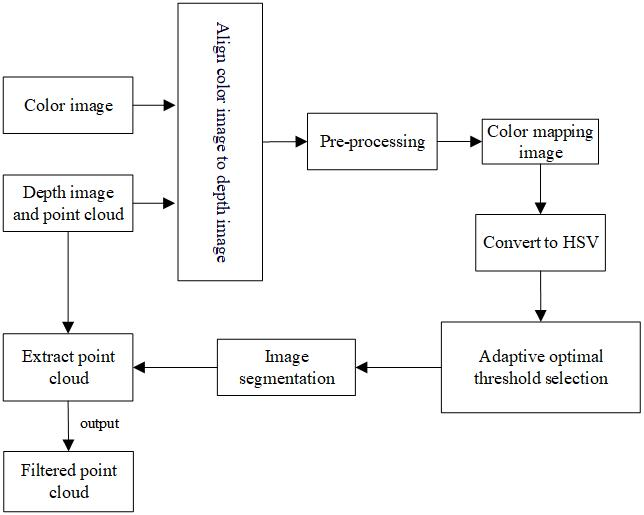

Supplement: S8 Fig — (TIF) [file pone.0220253.s008.tif]

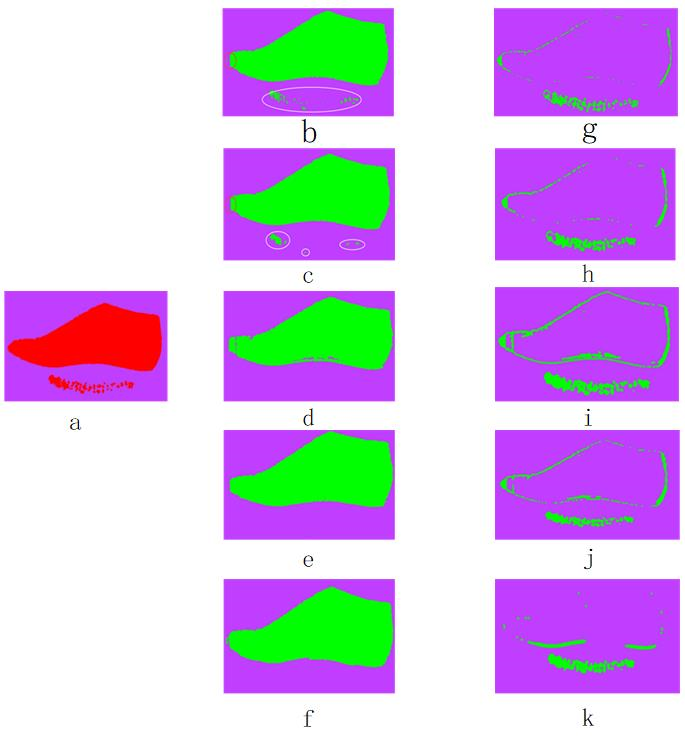

Supplement: S9 Fig — (TIF) [file pone.0220253.s009.tif]

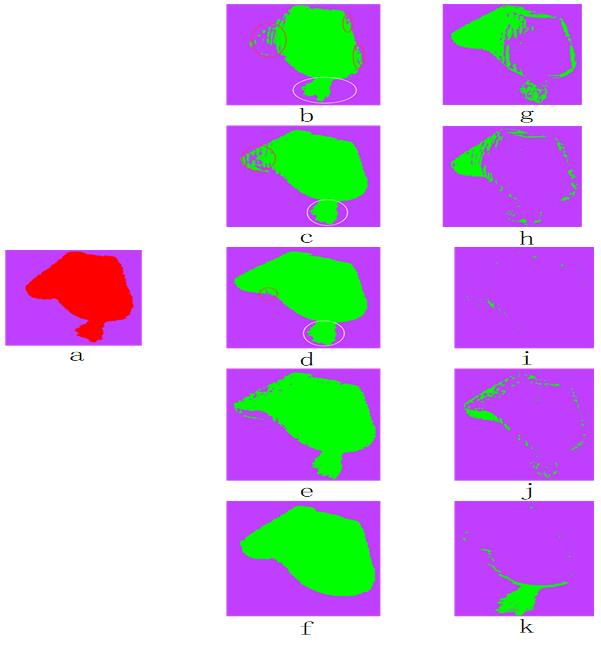

Supplement: S10 Fig — (TIF) [file pone.0220253.s010.tif]

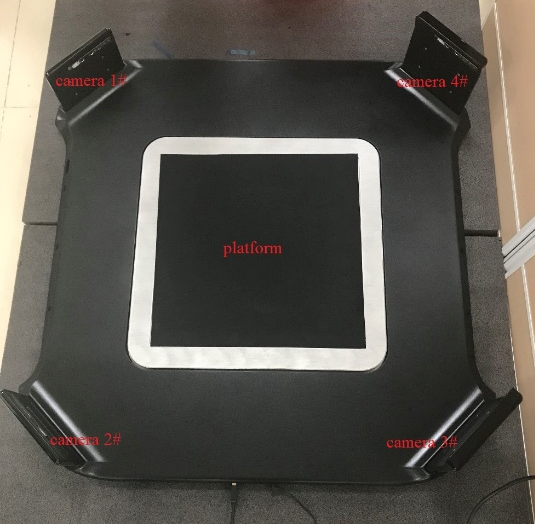

Supplement: S11 Fig — (TIF) [file pone.0220253.s011.tif]

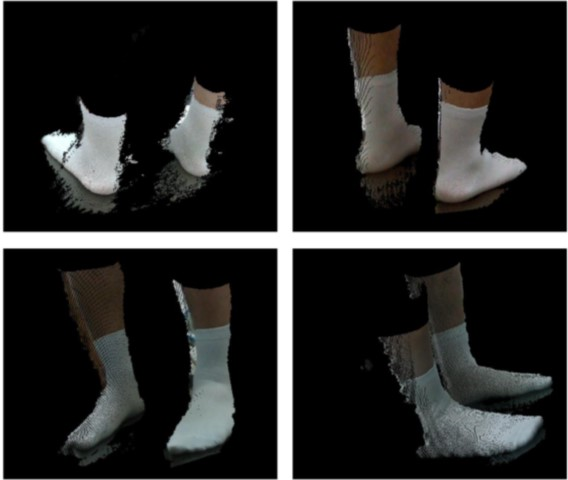

Supplement: S12 Fig — Scanning result: (a) Original point clouds. (TIF) [file pone.0220253.s012.tif]

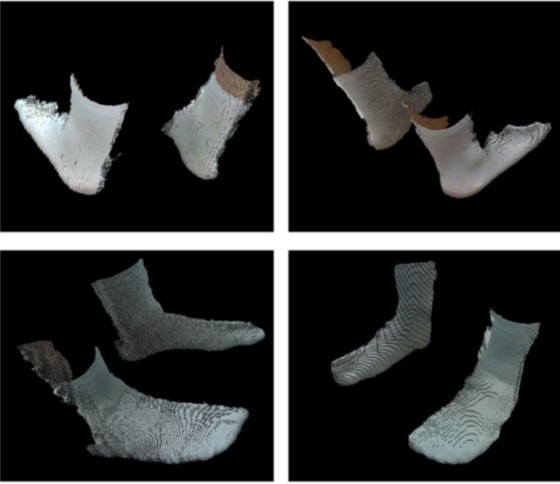

Supplement: S13 Fig — Scanning result: (b) Filtered point clouds. (TIF) [file pone.0220253.s013.tif]

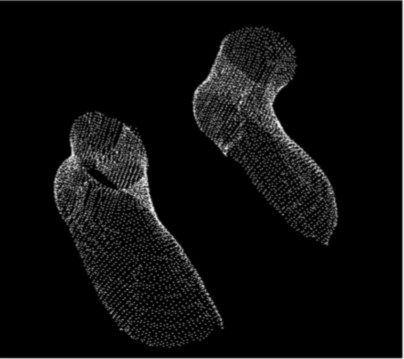

Supplement: S14 Fig — Scanning result: (c) Complete point cloud model. (TIF) [file pone.0220253.s014.tif]
